# Multi‐Domain Cognitive Assessment in a Scopolamine‐Induced Mouse Model: Toward a Translational Framework

**DOI:** 10.1002/alz70859_106326

**Published:** 2025-12-26

**Authors:** Jinhak Kim, Junho Kim, Euimo Yang, Jeong Yoon Lee, Ha Jin Jeong, Hyunjeong Kim, Keun You Kim, Eosu Kim

**Affiliations:** ^1^ Yonsei University College of Medicine, Seoul Korea, Republic of (South)

## Abstract

**Background:**

Dementia is a complex and heterogeneous condition, encompassing multiple subtypes and mixed pathologies that complicate translational research. Clinical assessments rely on neuropsychiatric evaluations to measure cognitive domains such as memory, attention, visuospatial function, executive function, and language. However, replicating these aspects in animal models remains a challenge. Implementing multi‐domain cognitive assessments in animal models is crucial for capturing the diverse cognitive impairments associated with dementia.

**Method:**

Male C57BL/6 mice were administered scopolamine to induce cognitive impairments, followed by treatment with donepezil. Cognitive functions were assessed using a touchscreen‐based operant system through the following tasks: Visual Discrimination (VD), 5‐Choice Serial Reaction Time Task (5‐CSRTT), Fixed/Progressive Ratio (FR/PR), and Paired Associate Learning (PAL).

**Result:**

In the VD task, scopolamine significantly reduced accuracy, confirming cognitive impairment, while donepezil reversed this effect, demonstrating the model’s sensitivity to treatment. In 5‐CSRTT, donepezil at 3 mg/kg effectively reversed scopolamine‐induced deficits, whereas 1.5 mg/kg was insufficient, indicating a dose‐dependent effect. In FR/PR, scopolamine increased the breakpoint, suggesting altered motivation, and donepezil did not reverse this effect, highlighting potential model limitations in assessing motivation‐related impairments. In PAL, scopolamine (1 mg/kg) reduced accuracy, confirming cognitive impairment in associative learning, but this effect was not reversed by donepezil.

**Conclusion:**

The scopolamine‐induced cognitive impairment model demonstrated domain‐specific deficits with task‐ and dose‐dependent treatment responses. These findings emphasize the need for optimization and task refinement to enhance model reliability. Structuring these tasks into a comprehensive multi‐domain battery could provide a robust preclinical platform for evaluating potential therapeutics and advancing our understanding of dementia’s cognitive complexities.

